# Protective antibody response following oral vaccination with microencapsulated *Bacillus Anthracis* Sterne strain 34F2 spores

**DOI:** 10.1038/s41541-020-0208-3

**Published:** 2020-07-10

**Authors:** Jamie S. Benn, Sankar P. Chaki, Yi Xu, Thomas A. Ficht, Allison C. Rice-Ficht, Walter E. Cook

**Affiliations:** 1 Texas A&M University, Department of Veterinary Pathobiology, College Station, TX 77843 USA; 2 Center for Infectious and Inflammatory Diseases, Institute of Biosciences and Technology, Texas A&M Health Science Center, Houston, TX 77030 USA; 3 Texas A&M Health Science Center, Department of Molecular and Cellular Medicine, College Station, TX 77843 USA

**Keywords:** Bacterial infection, Live attenuated vaccines, Live attenuated vaccines

## Abstract

An oral vaccine against anthrax (*Bacillus anthracis*) is urgently needed to prevent annual anthrax outbreaks that are causing catastrophic losses in free-ranging livestock and wildlife worldwide. The Sterne vaccine, the current injectable livestock vaccine, is a suspension of live attenuated *B. anthracis* Sterne strain 34F2 spores (Sterne spores) in saponin. It is not effective when administered orally and individual subcutaneous injections are not a practical method of vaccination for wildlife. In this study, we report the development of a microencapsulated oral vaccine against anthrax. Evaluating Sterne spore stability at varying pH’s in vitro revealed that spore exposure to pH 2 results in spore death, confirming that protection from the gastric environment is of main concern when producing an oral vaccine. Therefore, Sterne spores were encapsulated in alginate and coated with a protein shell containing poly-L-lysine (PLL) and vitelline protein B (VpB), a non-immunogenic, proteolysis resistant protein isolated from *Fasciola hepatica*. Capsule exposure to pH 2 demonstrated enhanced acid gel character suggesting that alginate microcapsules provided the necessary protection for spores to survive the gastric environment. Post vaccination IgG levels in BALBc/J mouse serum samples indicated that encapsulated spores induced anti-anthrax specific responses in both the subcutaneous and the oral vaccination groups. Furthermore, the antibody responses from both vaccination routes were protective against anthrax lethal toxin in vitro, suggesting that further optimization of this vaccine formulation may result in a reliable oral vaccine that will conveniently and effectively prevent anthrax in wildlife populations.

## Introduction

Anthrax infections have plagued humans and animals alike for millennia, possibly even causing the fifth and sixth plagues of Egypt^1^. The causative agent, *Bacillus anthracis*, has been studied since the beginning of microbiology but even after more than a century of scientific studies, the anthrax vaccination field has made little progress, especially with veterinary anthrax vaccines^1,2^. A recent study consolidated data from the last 20 years and found a worldwide distribution and suitability for anthrax in the environment with reports of the disease on every habitable continent, yet most animals remain unvaccinated^3^. While it may be prudent to mention that the incidence of human infection can be decreased with adequate livestock and wildlife vaccination policies, it should also be of great concern that free-ranging livestock and wildlife populations worldwide are unprotected against anthrax outbreaks that can cause catastrophic harm to sensitive wildlife conservation efforts^3–6^.

**Fig. 1:**
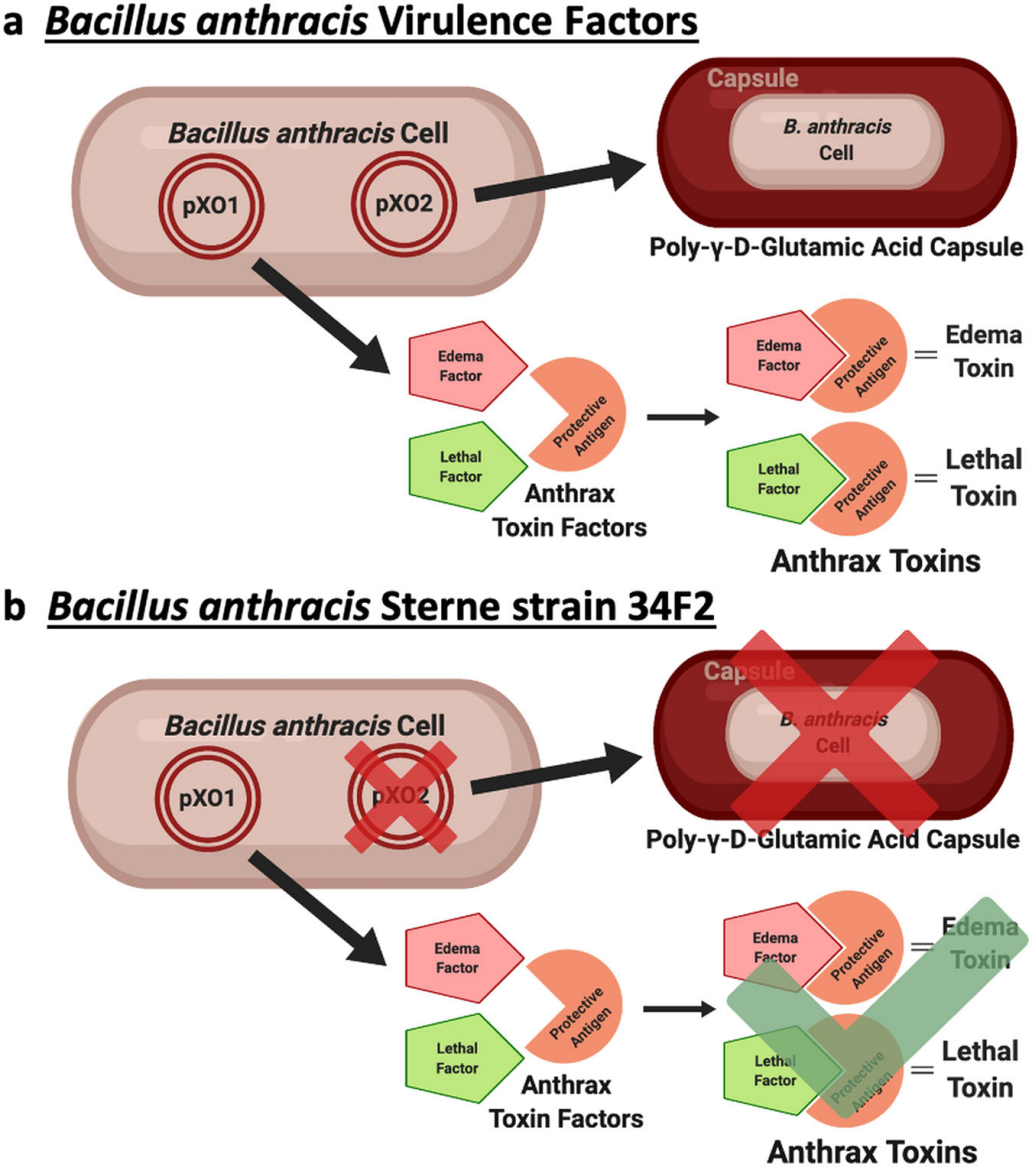
Illustration of *Bacillus anthracis plasmids*. **a** A fully virulent *B. anthracis* cell containing both the pXO1 and pXO2 plasmids. **b** *B. anthracis* Sterne strain 34F2 cell containing only the pXO1 plasmid. Created with BioRender.com.

The current veterinary vaccine, historically referred to as the Sterne vaccine, uses *B. anthracis* Sterne strain 34F2 spores (Sterne spores) that have naturally lost the pXO2 plasmid and therefore can no longer produce the poly-γ-D-glutamic acid capsule, also known as the anti-phagocytic capsule (Fig. 1)^6^. The original formulation of the Sterne vaccine, which is still in use today, consists of Sterne spores suspended in saponin and has been used to vaccinate domesticated livestock against anthrax since its discovery in the late 1930’s^1,7^. Despite decades of successful protections, the Sterne vaccine is outdated, impractical, known to vary in its potency, and can cause adverse reactions, occasionally even death^8^. Logistically speaking, the Sterne vaccine is distributed as a subcutaneous injection which is a highly impractical method of vaccination for free-ranging livestock and wildlife^1^. Without a reasonable method of wildlife vaccination, yearly anthrax outbreaks in national parks and other wildlife areas worldwide pose economic, ecological and conservational burdens to wildlife and wildlife health professionals^3,7,9,10^. Even with these yearly outbreaks, the anthrax spore distribution in these areas is undetermined so it isn’t possible to vaccinate wildlife based on an estimated risk of exposure^11^. The most feasible way to protect wildlife in these areas would be via oral vaccination however, after results from a previous study demonstrated that the Sterne vaccine is incapable of eliciting an immune response following oral vaccination, the urgent need for an effective oral anthrax vaccine for wildlife has never been more evident^12^.

Other research groups in the oral anthrax vaccination field have reported encouraging results from vaccines expressing a recombinant form of anthrax protective antigen in a variety of bacterial, viral or plant-based expression systems^13–16^. Any progress toward the development of an oral anthrax vaccine is a great achievement, but studies have suggested that anthrax spore associated antigens may also make important contributions to vaccine induced protection^17,18^. Since it is possible that exposure to a single recombinant antigen may not stimulate sufficient immune activity to protect against fully virulent exposure^17^, it may be advantageous to maintain the live attenuated format of the Sterne vaccine, but adapt it for oral use.

One method to accomplish this is through the exceptionally high flexibility of alginate encapsulation^19–21^. Alginate is naturally indigestible in mammalian systems which can be implemented as a natural controlled release vehicle^22,23^. In addition, the mild gelation conditions permit entrapment of the desired capsule load without significantly affecting the viability^22^. Post-gelation, the viability of the capsule load is maintained by stability of the microcapsule, particularly in gastric environments which has proven overwhelmingly beneficial for the development of probiotics^20^. Alginate has also demonstrated bio-adhesive properties when interacting with mucosal tissues. Combined with the depot effect of alginate capsules, these bio-adhesive properties ensure that the capsule load is repeatedly released in close proximity to target cells^19^.

The beneficial characteristics of alginate microcapsules can also be specifically tailored to each application by altering the capsule size, structure, load, layers, and many other aspects of each capsule formulation. When it comes to oral vaccine delivery incorporating stabilizing components, such as poly-L-lysine (PLL) and vitelline protein B (VpB), into the alginate microcapsule formulation can enhance the overall capsule stability. PLL is a common microcapsule coating that stabilizes alginate cross-linking^24–26^. VpB, a non-immunogenic, eggshell precursor protein isolated from the parasite *Fasciola hepatica*, is resistant to enzymatic and chemical degradation and can extend the already slow erosion of the alginate capsule^27,28^. Prior studies from our research group have applied a protein shell containing both of these components to microencapsulated *Brucella spp*. and reported stronger, extended immune responses that correlated with enhanced protection against wild-type challenge^29–32^.

In the current study, we evaluated the effect that this formulation had on the stability of microcapsules as enteric delivery vehicles. We also examined the immunogenicity of microencapsulated Sterne spores and observed a pronounced increase in the resulting antibody response from both subcutaneous and oral vaccination in mice. Moreover, an in vitro toxin challenge revealed that the observed antibody response was protective for the macrophage cell line following oral vaccination suggesting that with further optimization, microencapsulated Sterne spores can be developed into an alternative anthrax vaccine formulation capable of efficient and protective vaccination of free-ranging livestock and wildlife.

## Results

### Sterne spore stability in simulated gastrointestinal environments

**Fig. 2:**
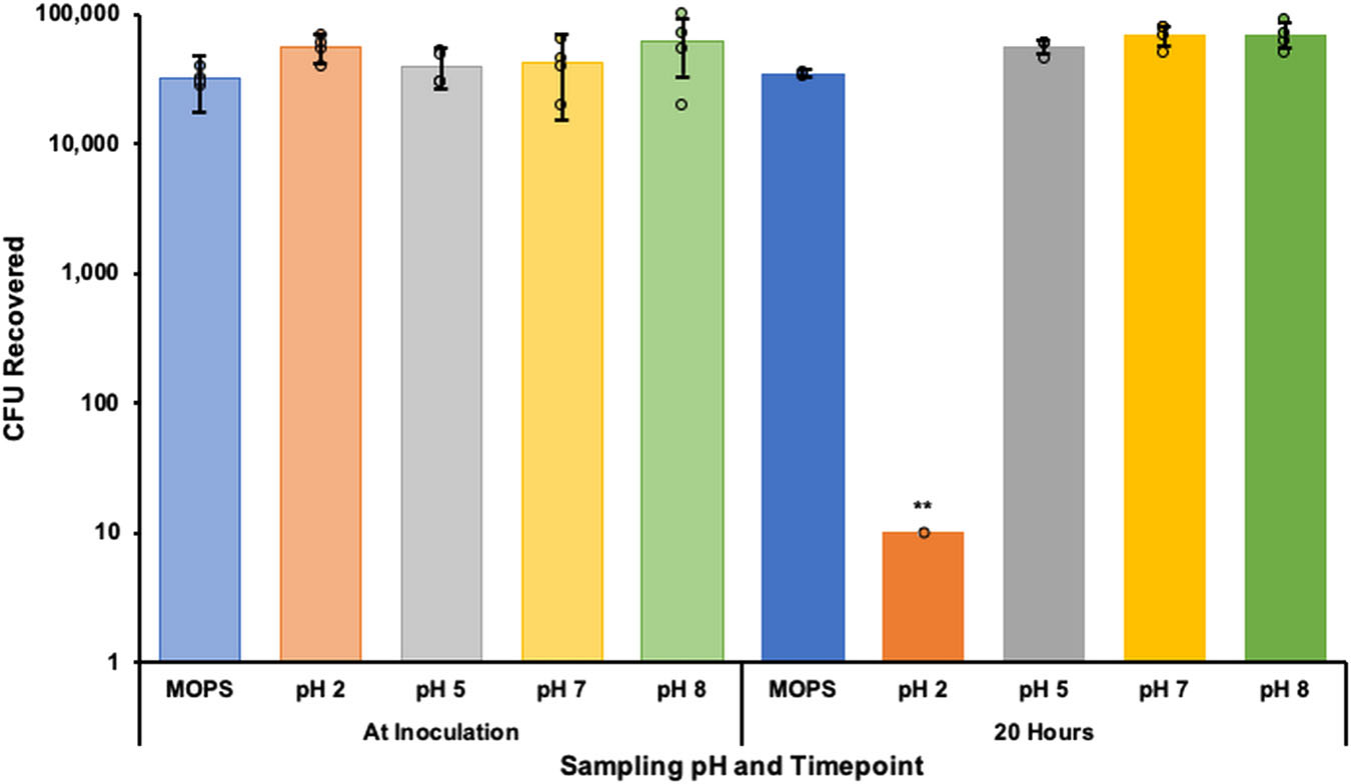
Sterne spore titer response to simulated gastrointestinal environments. Simulated gastric (0.2% (w/v) NaCl, pH 2 and pH 5) and intestinal (0.68% (w/v) KH_2_PO_4_, pH 7 and 8) fluids were inoculated with 6.8 × 10^5^ *Bacillus anthracis* Sterne strain 34F2 spores and incubated overnight at 37 °C with shaking. MOPS buffer (10 mM MOPS, 0.85% NaCl) was also inoculated with 6.8 × 10^5^ Sterne spores to serve as a negative control for encapsulated vaccine storage conditions. The resulting viable bacterial titer in each solution was determined by plating serial dilutions. Differences between starting and resulting titers were determined by Student’s *t*-tests with **p < 0.01.

The unencapsulated Sterne spore response to simulated gastrointestinal fluids (GI fluids) was observed to better understand and account for impairments while in transit through the stomach and intestines. Simulated gastric (0.2% (w/v) NaCl, pH 2 and pH 5) and intestinal (0.68% (w/v) KH_2_PO_4_, pH 7 and 8) fluids^33^ were inoculated with 6.8 × 10^5^ *Bacillus anthracis* Sterne strain 34F2 spores and incubated overnight at 37 °C with shaking. MOPS buffer (10 mM MOPS, 0.85% NaCl, [pH 7.4]) was also inoculated with 6.8 × 10^5^ Sterne spores to serve as a negative control for encapsulated vaccine storage conditions. The unencapsulated Sterne spore titer was severely reduced as a result of exposure to 0.2% NaCl (w/v) pH 2 (*p* < 0.01) with no other significant responses observed from pH 5, 7 or 8 (Fig. 2).

### Comparison of microcapsule formulations in gastrointestinal environments

**Fig. 3:**
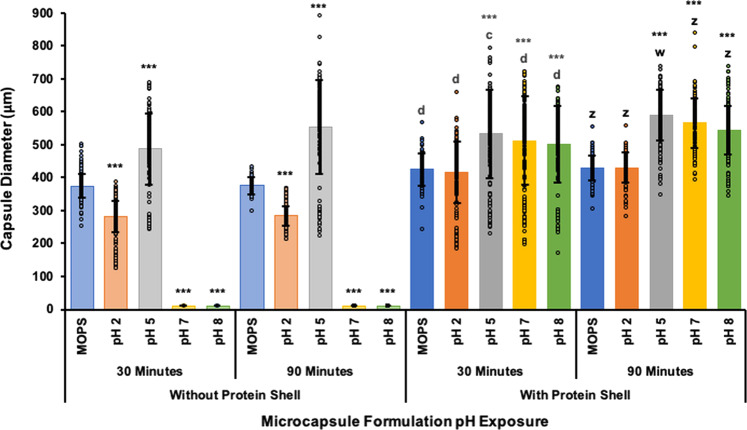
Microcapsule response to simulated gastrointestinal fluids. Microcapsules with and without protein shell were suspended in simulated gastric (0.2% (w/v) NaCl, pH 2 and pH 5) and intestinal (0.68% (w/v) KH_2_PO_4_, pH 7 and 8) fluids for 30 and 90 min at 37 °C with shaking. Microcapsule samples were also suspended in MOPS buffer (10 mM MOPS, 0.85% NaCl) as a negative control for encapsulated vaccine storage conditions. The capsule diameters after exposure to simulated gastrointestinal fluids were observed in brightfield and measured in ImageJ. Data are reported as the average capsule diameter for the group in μm ± the standard deviation and changes were determined by one-way ANOVA followed by the Tukey–Kramer HSD test. Significant differences from pre-exposure diameters in MOPS within the same group are identified as ***p < 0.001. Differences between exposure diameters in microcapsules with and without the protein shell after the 30-min incubation are identified with c, *p* < 0.001 and d, *p* < 0.0001. Differences between exposure diameters in microcapsules with and without the protein shell after the 90-min incubation are identified with w, *p* < 0.05 and z, *p* < 0.0001.

**Fig. 4:**
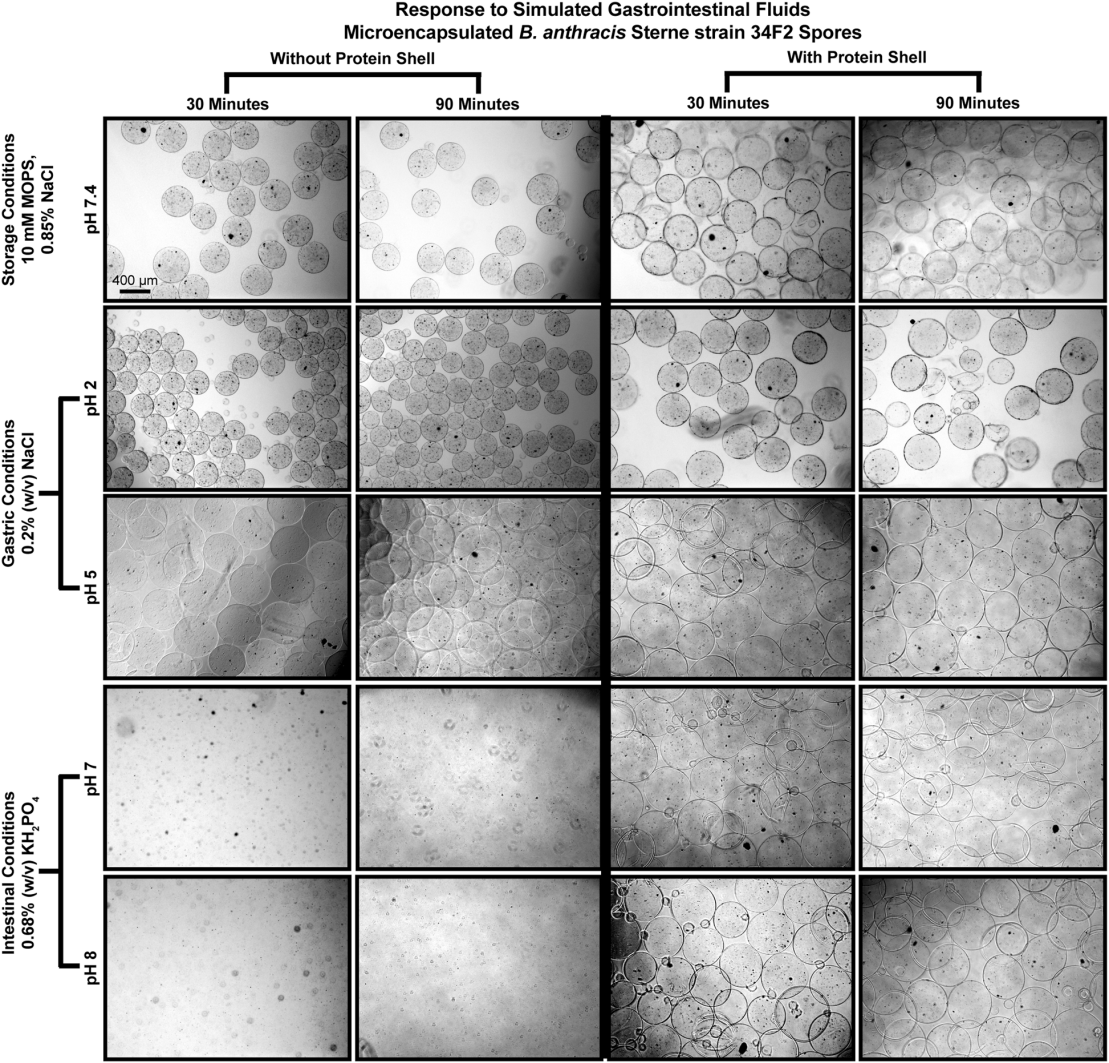
Microcapsule response with and without the protein shell to simulated gastrointestinal environments. Representative brightfield images of microcapsule samples following exposure to simulated gastric (0.2% (w/v) NaCl, pH 2 and pH 5) and intestinal (0.68% (w/v) KH_2_PO_4_, pH 7 and 8) fluids for 30 and 90 min at 37 °C with shaking. Microcapsule samples were also suspended in MOPS buffer (10 mM MOPS, 0.85% NaCl) as a negative control for encapsulated vaccine storage conditions. The bottom two images of the two left columns portray the dissolution of capsules without the protein shell at pH 7 and pH 8. Bar represents 400 μm.

Microcapsules were also exposed to GI fluids to observe the relative stability in simulated gastrointestinal conditions^33^ with and without the PLL and VpB shell (protein shell). Microcapsule samples were suspended in MOPS buffer as a negative control and simulated GI fluids at pH 2, 5, 7, and 8 for 30 and 90 min at 37 °C with shaking. At pH 2, capsules that were not coated with the protein shell were shown to decrease in diameter compared to neutral storage conditions in MOPS, whereas at pH 5 capsules without the protein shell experienced significant swelling (Fig. 3). The most striking advantage of the protein shell was its capsule stabilization abilities at pH 7 and 8. Without the addition of this proteolysis resistant coating, the capsules completely disintegrated at neutral pHs (Fig. 4). These patterns were also observed in uncoated capsules after 90 min in GI fluids, simply to a higher degree as a result of the extended exposure. In comparison, capsules with the protein shell exhibited overall enhanced stability in all GI fluids by preventing shrinking at pH2 and complete capsule dissolution at pH 7 and 8 (Fig. 4).

### Evidence of bacterial entrapment and controlled release from microcapsules

**Fig. 5:**
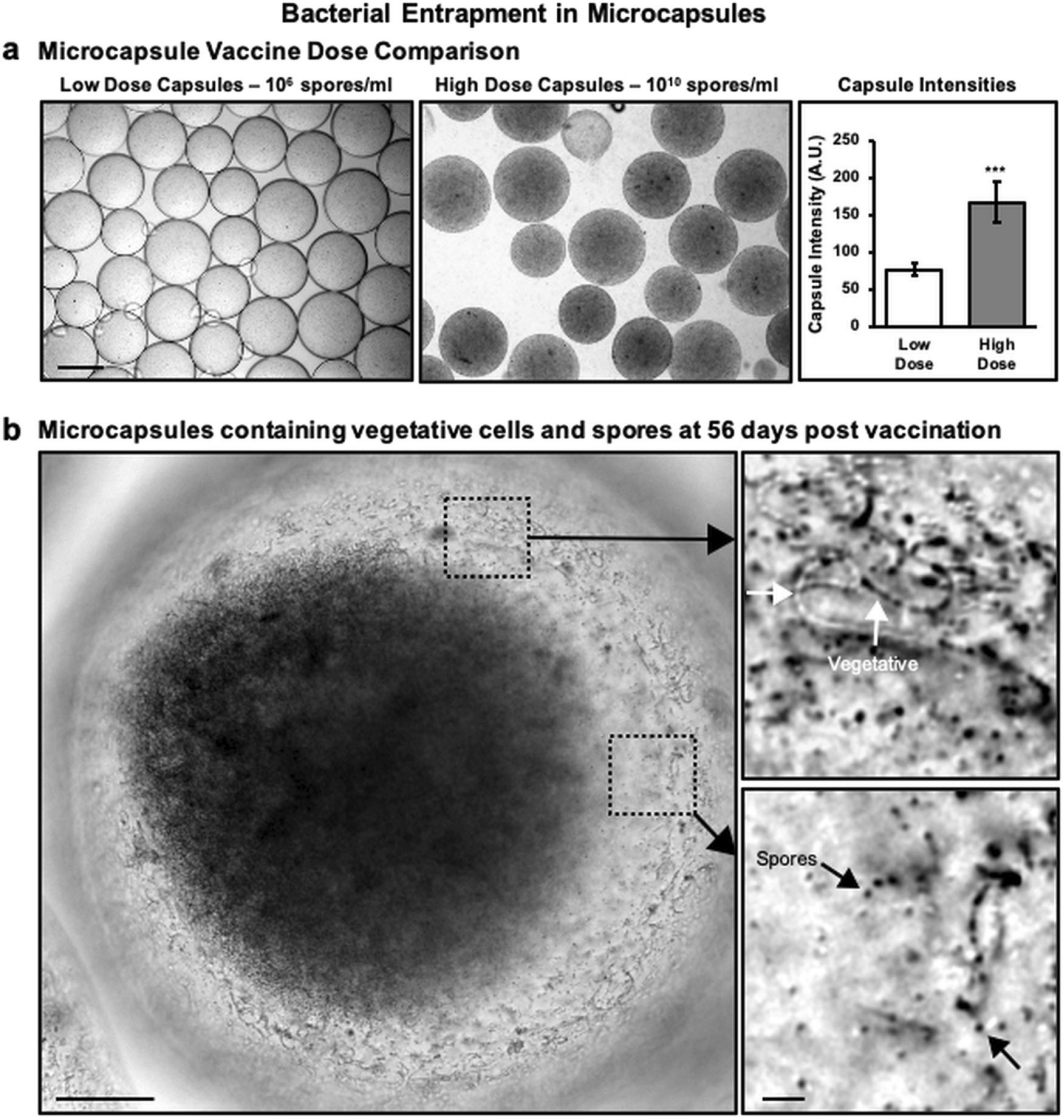
Sterne spore entrapment in microcapsules. **a** Visual and pixel intensity comparison between Low and High Dose Capsules demonstrates the difference (***p < 0.0001) between the encapsulated doses as determined by Student’s *t*-tests. Bar represents 400 μm. **b** A considerable amount of aggregated Sterne cells are still entrapped within the microcapsules, as seen in this close up image of a single High Dose microcapsule 56 days after starting the in vitro release experiment. Bar represents 100 μm (left). Magnified images of vegetative cells (top right) and spores (bottom right) that remain entrapped within the High Dose microcapsule. Bar represents 10 μm.

**Fig. 6:**
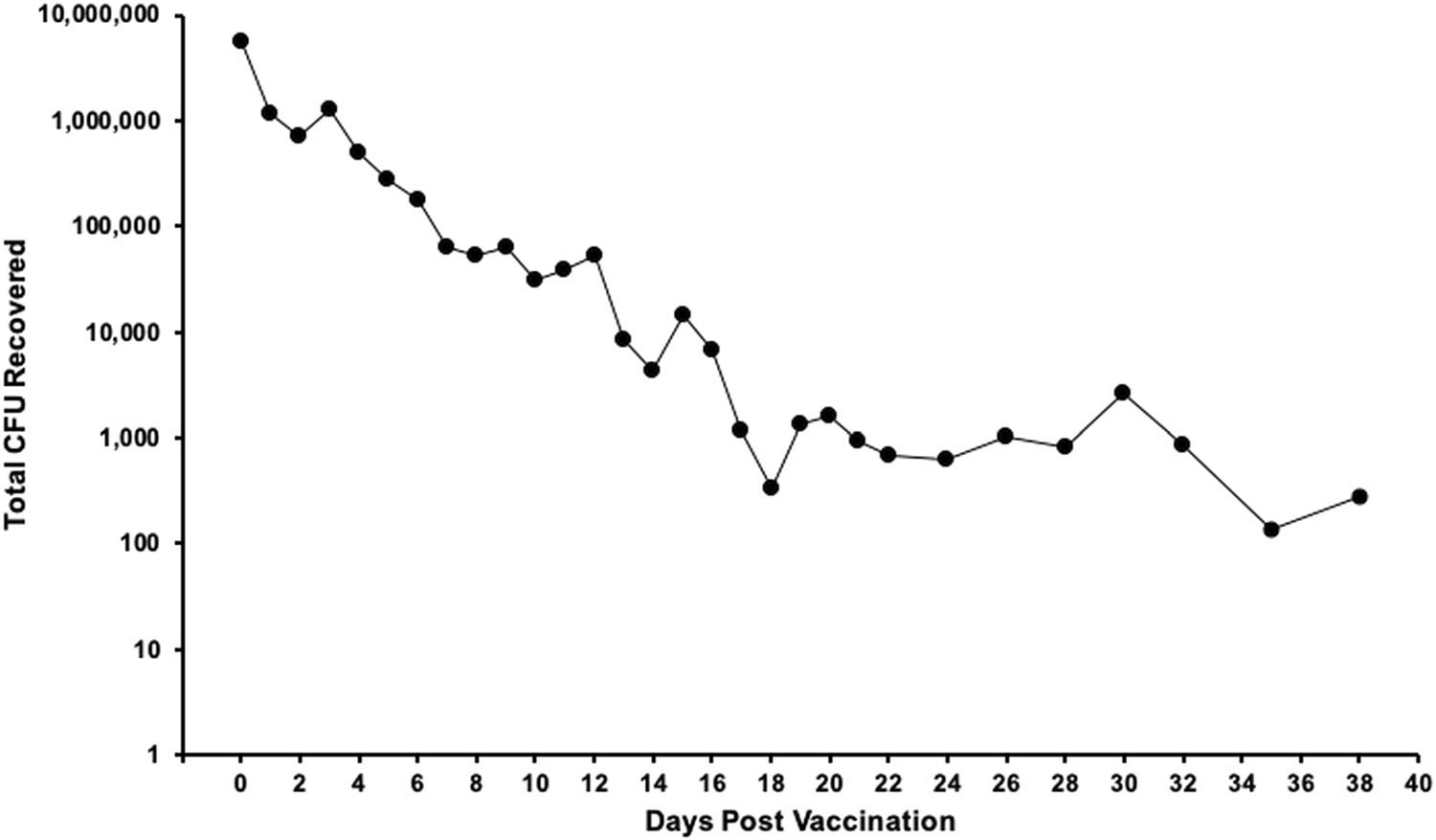
In vitro bacterial release from microcapsules. A 1 ml sample of microcapsules with the protein shell was suspended in 10 ml MOPS at 37 °C with shaking. The MOPS buffer was completely removed and replaced each day. The collected supernatant was serially diluted and plated onto LB agar to quantify the CFU that had been released each day.

Microcapsules were imaged in the brightfield to confirm ideal capsule formation and bacterial entrapment. The drastic increase in the amount of encapsulated Sterne spores is visible when comparing the Low Dose Capsules with the High Dose Capsules in storage conditions which were made with 5 × 10^6^ spores/ml and 4 × 10^10^ spores/ml, respectively (Fig. 5a, left and middle). This significant increase (*p* < 0.0001) is also evidenced by measuring the pixel intensity of the microcapsule images (Fig. 5a, right). An in vitro release experiment was conducted by collecting samples for 38 days to evaluate the timeframe of bacterial release from microcapsules coated with the protein shell. Microcapsules were suspended in 1 ml of MOPS buffer and incubated at 37 °C with shaking. The supernatant was removed and replaced at frequent intervals then serially diluted on LB agar to quantify the release rate. Results depicted in Fig. 6 confirm the sustained release abilities of microcapsules coated with the protein shell. Although the daily sample collection was stopped beyond 38 days, the full experiment was terminated at the same time as the mouse immunization experiment on day 56 when a final bacterial release sample and the remaining capsules were collected for imaging. After 56 days of shaking at 37 °C, the capsules still contained aggregations of viable Sterne spores and vegetative cells (Fig. 5b) suggesting that capsules would have been able to continue releasing viable bacteria for much longer.

### Microcapsule vaccines induce anthrax specific antibody responses

**Fig. 7:**
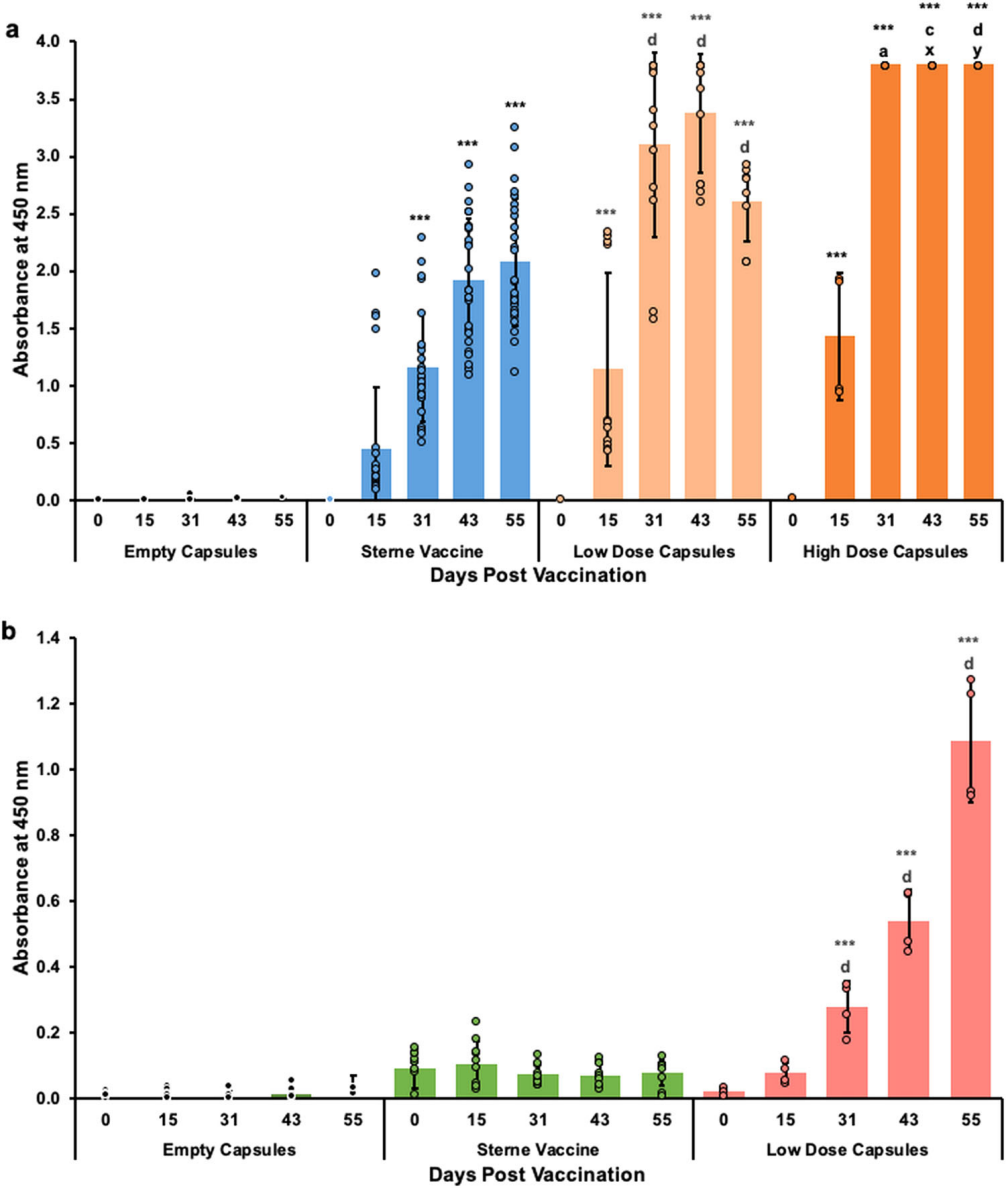
IgG responses from subcutaneous and oral vaccination with Empty Capsules, Sterne vaccine, Low Dose Capsules and High Dose Capsules. BALBc/J mice were either subcutaneously injected (**a**) or orally inoculated (**b**) with 10^6^ unencapsulated *B. anthracis* Sterne strain 34F2 spores or 10^6^ encapsulated Sterne spores in Low Dose Capsules. An additional group of mice were subcutaneously injected with 10^9^ encapsulated Sterne spores in High Dose Capsules (**a**). All capsule vaccines were coated with the protein shell. The control groups received Empty Capsules. Serum samples were collected at 0, 15, 31, 43- and 55-days post vaccination and analyzed by ELISA. Antibody responses were analyzed by one-way ANOVA followed by the Tukey–Kramer HSD test and are shown as mean absorbances at 450 nm ± standard deviation from the 1:2000 dilution for subcutaneously inoculated mice and from the 1:125 dilution for orally inoculated mice. Significant differences from pre-vaccination (Day 0) within the same group are identified as ***p < 0.0001. Differences between responses to the Sterne vaccine and Low Dose Capsules at corresponding time points are identified with a, *p* < 0.05; c, *p* < 0.001 and d, *p* < 0.0001. Differences between responses to the Low Dose Capsules and High Dose Capsules at corresponding time points are identified with x, *p* < 0.01 and y, *p* < 0.0001.

Antibody levels against anthrax protective antigen were measured by end point ELISA. All vaccines containing Sterne spores elicited strong antibody responses starting at 15 days post subcutaneous vaccination (Fig. 7a, Supplementary Figs. 1–3, Supplementary Table 1). Despite being inoculated with the same dose of spores (Table 1), the Low Dose Capsule group demonstrated a higher antibody response than the Sterne vaccine group on days 31 and 43 (Fig. 7a, Supplementary Figs 1 and 2, Supplementary Table 1). These antibody levels were even further increased in mice that were subcutaneously vaccinated with the High Dose Capsules which resulted in higher IgG titers at all time points (Fig. 7a, Supplementary Fig. 3, Supplementary Table 1). A similarly improved antibody response was also observed from the orally administered Low Dose Capsules starting at 31 days post vaccination and it continued to increase each week (Fig. 7b, Supplementary Fig. 4, Supplementary Table 1). Both orally administered vaccines contained the same dose of Sterne spores, but the oral Sterne vaccine did not induce any antibody response (Fig. 7b, Supplementary Fig. 5).

**Table 1:** Vaccination groups to assess the efficacy of microencapsulated Sterne spores as an oral vaccine.

Route	Group (*n* = 5)	Inoculation volume	Spores/ml	Spores/mouse	Blood collection (days post-vaccination)
SC	Empty Capsules	0.2 ml	–	–	0, 15, 31, 43, 55
SC	Sterne vaccine	0.2 ml	5 × 10^6^	1 × 10^6^	0, 15, 31, 43, 55
SC	Low Dose Capsules	0.2 ml	5 × 10^6^	1 × 10^6^	0, 15, 31, 43, 55
SC	High Dose Capsules	0.2 ml	4 × 10^10^	9 × 10^9^	0, 15, 31, 43, 55
Oral	Empty Capsules	0.2 ml	–	–	0, 15, 31, 43, 55
Oral	Sterne vaccine	0.2 ml	5 × 10^6^	1 × 10^6^	0, 15, 31, 43, 55
Oral	Low Dose Capsules	0.2 ml	5 × 10^6^	1 × 10^6^	0, 15, 31, 43, 55

*SC* subcutaneous, *Empty capsules* Microcapsules with PLL and VpB shell (no bacteria), *Sterne vaccine* *B. anthracis* Sterne strain 34F2 spores in saponin, *Low Dose Capsules* microcapsules with the protein shell and the standard dose of Sterne spores, *High Dose Capsules* microcapsules with the protein shell and a higher dose of Sterne spores.

### Microencapsulated Sterne spores induce toxin neutralizing antibodies

**Fig. 8:**
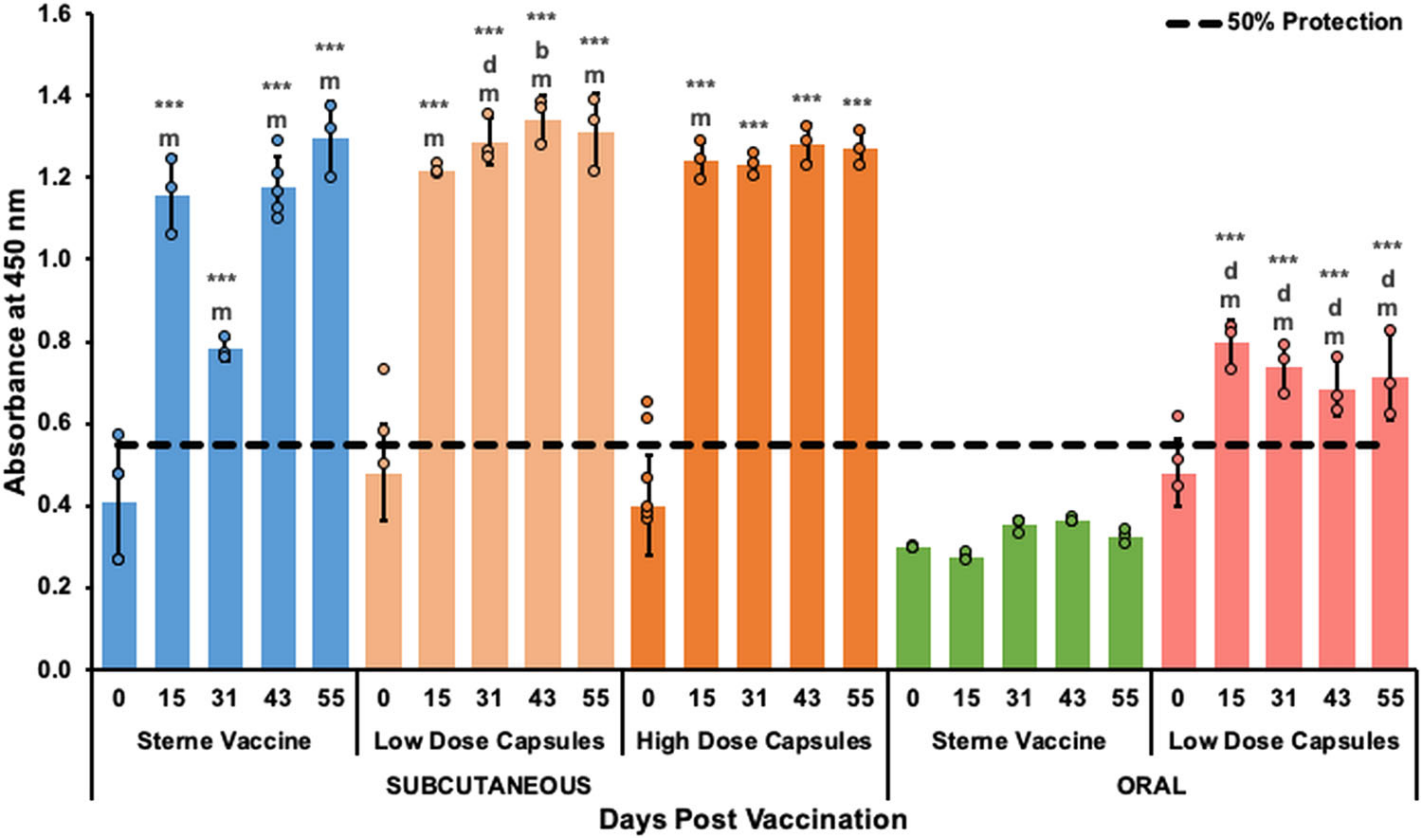
In vitro toxin neutralizing abilities of antibodies from subcutaneous and oral vaccination with the Sterne vaccine, Low Dose Capsules and High Dose Capsules. Serum was collected from mice at 0, 15, 31, 43- and 55-days post subcutaneous or oral vaccination with 10^6^ unencapsulated *B. anthracis* Sterne strain 34F2 spores, 10^6^ encapsulated Sterne spores in Low Dose Capsules or 10^9^ encapsulated Sterne spores in High Dose Capsules. Both Low and High capsule vaccines were coated with the protein shell. Control groups received Empty Capsules (results not included in this graph). Diluted serum samples were pre-incubated with LeTx then added to J774A.1 cells and the resulting cell viability was assessed with MTT dye. Data presented here represent the average absorbance at 595 nm ± standard deviations for each group at each time point at a 1:50 dilution with differences determined by one-way ANOVA followed by the Tukey–Kramer HSD test. Significant differences from pre-vaccination (Day 0) within the same group are identified as ***p < 0.0001. Differences between the Sterne Vaccine and Low Dose Capsules at corresponding time points are identified with b, *p* < 0.01 and d, *p* < 0.0001. Differences between subcutaneous and oral vaccination responses with the same vaccines at corresponding time points are identified with m, *p* < 0.0001. Differences between responses to the Low Dose Capsules and High Dose Capsules at corresponding time points were not significant.

Lethal Toxin (LeTx) neutralization assays evaluated the ability for vaccination induced antibody responses to protect J774A.1 cells from LeTx mediated killing. The toxin neutralizing abilities of all vaccination groups are presented in Fig. 8 as mean absorbances at 595 nm ± the standard deviation at a single serum dilution of 1:50. Neutralizing antibody titers were estimated with serial dilutions and are reported in Supplementary Table 2. In agreement with the ELISA results, serum from all subcutaneously injected encapsulated vaccines containing Sterne spores appear to prevent ~100% of LeTx induced mortality in vitro at all measured time points (Fig. 8). The Low Dose Capsule vaccine exhibited enhanced LeTx neutralizing abilities at 31, 43, and 55-days post vaccination with stronger improvements induced by the High Dose Capsule vaccine (Supplementary Table 2). Strikingly, the orally administered Low Dose Capsule vaccine also resulted in partial toxin neutralizing effects at the same dilution as subcutaneously immunized mice (Fig. 8). Serum from mice immunized orally with the Sterne Vaccine did not provide any protection from LeTx challenge in vitro (Fig. 8, Supplementary Table 2).

## Discussion

The benefits of oral vaccine delivery cannot be overstated, particularly when it comes to protecting free-ranging livestock and wildlife from current and emerging infectious diseases such as anthrax. Development of oral vaccines can allow for easy, wide-spread vaccination policies without needing to deal with the labor-intensive programs and painful injections associated with the majority of today’s human and animal vaccines. It is also possible that effective oral vaccines may be intrinsically more stable and have longer shelf-lives as a collateral benefit of the stability required for transit through the gastrointestinal tract. Furthermore, oral vaccines can lead to enhanced efficacy with less adverse effects due to mucosal immunity and oral delivery.

For all of these reasons and more, an alternative anthrax vaccine formulation specifically for oral administration is urgently needed to protect animals worldwide from potentially catastrophic anthrax outbreaks^3,12^. Many wildlife health professionals have demanded a new veterinary anthrax vaccine because individual hand-injections for each and every animal is not a practical method of vaccination for wildlife and a recent study demonstrated that oral vaccination with the Sterne vaccine is not effective^1,12^. Also, sustained protection from the Sterne vaccine can only be achieved with annual boosters which requires a yearly cycle of troublesome injections with the potential for adverse reactions^1^. To resolve the many issues associated with anthrax outbreaks and vaccination, we developed and evaluated an innovative anthrax vaccine formulation for oral vaccination. Results of our study demonstrate that subcutaneous and oral vaccination with microencapsulated *B. anthracis* Sterne strain 34F2 spores can induce antibody production in the murine model which can inactivate *B. anthracis* lethal toxin in vitro.

Oral vaccination is a common goal throughout the entire vaccinology field but there are still a limited number of oral vaccines approved for animal and human use because the main obstacle facing oral vaccination is, ironically, oral vaccination itself^34–36^. The principle of oral vaccination is completely dependent on getting sensitive antigens through the harsh, gastric environment that was evolutionarily designed specifically to prevent that exact thing from happening. In contrast, gastrointestinal pathogens, such as anthrax, have also evolved over thousands of years to survive the gastric environment for eventual uptake in the small intestine but these pathogen survival strategies aren’t typically conserved in live attenuated organisms, our most reliable vaccine format. Such is the case with *B. anthracis* Sterne strain 34F2. Upon exposure to a simulated gastric environment, there was a severe decrease in the viable Sterne spore titer (Fig. 2). Given that the majority of anthrax infections in wildlife are gastrointestinal, it can be reasoned that fully virulent anthrax spores are able to survive passage through a harsh acidic environment to establish infections following uptake in the small intestine. In comparison to the experiments performed here with the pXO2-negative Sterne strain, this suggests that fully virulent anthrax spores may be better equipped to survive the gastrointestinal environment due to retention of the pXO2 plasmid (Fig. 1). The details of this hypothesis were not investigated in this study but may be an important avenue to consider as we strive to understand and prevent infection with this pathogen. Sterne strain vulnerability to the simulated gastric environment also implies that development of an oral vaccine with the Sterne strain must involve some protection to ensure safe passage through the stomach. In the current study, we demonstrate that alginate encapsulation with a proteolysis resistant protein shell is able to shield Sterne spores enough through the gastric environment to induce an immune response following oral vaccination.

We assessed the stabilizing and shielding abilities of the microcapsules produced in this study by observing the microcapsule responses to simulated gastrointestinal environments. When alginate capsules are formed in a cross-linking solution, guluronate residues in the alginate cooperatively bind Ca^2+^ ions from the solution, thus cross-linking the alginate polymers to the “pre-gel” state^21,24^. Exposure of a calcium cross-linked pre-gel to nongelling cations, such as Na^+^, will reduce the mechanical stability of the alginate gel and possibly disintegrate the entire polymer matrix, as exhibited in Fig. 4^21,25^. This can be prevented by adding additional cross-linked layers to the microcapsules, thus resulting in more stable capsules which we have demonstrated here by exposing uncoated and coated microcapsules to gastrointestinal environments^37^.

The added stability of these layers can be assessed through changes in microcapsule shrinking, swelling, and overall morphology. Changes in the alginate polymer network such as these can greatly affect the rate of diffusion through and the erosion of the network, thereby altering the antigen release rate^22,38,39^. Capsule shrinkage, as observed in uncoated capsules at pH 2 (Fig. 3), is indicative of increased acid-gel strength under higher proton concentrations^33^. Conversely, suspending uncoated capsules in pH 5 caused excessive swelling which implies increased pore size and therefore release of bacteria, whereas pH 7 and pH 8 resulted in complete disintegration of uncoated alginate capsules in the presence of nongelling ions (Fig. 4)^21,25^. Results of this study demonstrate the efficacy of using the PLL and VpB protein shell in this microcapsule formulation because it prevented most of the destabilizing affects observed in uncoated capsules. Specifically, the protein shell reduced the degree of swelling experienced by the capsules at pH 5, thereby avoiding drastic changes in the polymer network that could have led to premature bacterial release. Of most importance was that the protein shell maintained the capsule integrity at pH 7 and 8, whereas other studies have observed alginate disintegration at pH 7 and 8^40,41^. By preventing complete capsule dissolution in neutral environments, the protein shell ensures that the capsule is stable enough to serve its controlled release purpose by stimulating mucosal immunity and uptake in the intestines. While it would have also been valuable to determine the surviving encapsulated spore titer following uncoated and coated capsule exposure to gastrointestinal pHs, this would have involved dissolving the coated capsules in trypsin which likely would have had its own effect on the viable Sterne spore recovery, therefore confounding any results that may have been acquired from the experiment.

A second challenge to oral vaccination, after having endured the harsh gastric environment, is to ensure antigen transport across the intestinal epithelia followed by antigen-presenting cell activation^36^. Advances in particulate vaccine delivery vehicles suggest that both issues can be resolved with microencapsulation. Prior studies have reported improved bacterial stability under acidic conditions due to microencapsulation in alginate^20,21,42–44^. In addition, alginate capsules can improve antigen uptake and processing by antigen-presenting cells through the depot effect^45,46^. These advantages proved beneficial in previous studies from our laboratory when microencapsulation of *Brucella spp*. in alginate microcapsules coated with the protein shell increased immune responses and reduced challenge organism recovery following oral vaccination^29–32^. Similar enhancements were observed in this study when we applied the same encapsulation method to *B. anthracis* Sterne strain 34F2 spores.

Subcutaneous vaccination with Low Dose Capsules enhanced the observed antibody response even though mice received the same dose of spores as those vaccinated with the Sterne vaccine (Fig. 7a, Supplementary Figs 1 and 2, Supplementary Table 1). Increasing the encapsulated spore dose also resulted in an even more robust antibody response following subcutaneous vaccination with High Dose Capsules (Fig. 7a). Excitingly, ELISA results also revealed a significant improvement in the amount of antibody produced following oral vaccination with the Low Dose Capsules when compared to the Sterne vaccine (Fig. 7b). Although this response is still lower than that of the subcutaneously injected vaccines, to our knowledge this is the first time a measurable antibody response has ever been recorded following oral vaccination with live attenuated Sterne spores. The single prior attempt we are aware of involved mixing Sterne spores with scarifying agents for oral vaccination by way of tiny lacerations in the gums, tongue, oropharynx, etc. and observed limited success^47^. In contrast, results presented here were obtained from mice vaccinated by oral gavage which completely bypassed the oral mucosa. This suggests that microencapsulation with the protein shell provides enough protection for Sterne spores to survive the gastric environment and progress into the small intestine to stimulate an immune response.

The advantages of this microcapsule formulation were also detected in results from toxin neutralization assays (Fig. 8) which are considered an additional marker and stronger correlate of protection^13–15,48,49^. Subcutaneous vaccination with Low Dose Capsules resulted in better protection for cultured macrophages at 31, 43, and 55-days post vaccination when compared to the unencapsulated Sterne vaccine (Supplementary Table 2). In addition, subcutaneous injection with ~9 × 10^9^ Sterne spores per mouse in High Dose Capsules resulted in extraordinarily high serum IgG responses (Fig. 7a) that were fully protective in vitro by 15 days post vaccination (Fig. 8, Supplementary Table 2). This antibody response also may not yet have reached its peak prior to the end of the experiment. The in vitro release experiment demonstrated the controlled release abilities of this microcapsule formulation over the first 38 days (Fig. 6). However, images obtained from this experiment also showed that there was an excessive amount of Sterne spores and vegetative cells still entrapped within the high-dose capsules 56 days after vaccination suggesting that the controlled release could have continued for much longer (Fig. 5b).

According to previous work on mouse susceptibility to *B. anthracis* strains, the LD_50_ for BALB/cJ mice subcutaneously injected with the Sterne strain was 6.8 × 10^7^ spores^50^. In this study, BALBc/J mice were subcutaneously injected with over 100-fold times more Sterne spores with only one death, implying that this microencapsulation method can allow for enhanced protection with higher Sterne spore doses and less reactogenicity. Inoculation with a higher dose of Sterne spores could also be critical for successful oral vaccination. Sterne spore exposure to acidic environments greatly reduces the viable spore titer (Fig. 2), so vaccinating with a higher dose of microencapsulated Sterne spores may account for any titer loss due to the gastric environment^20^.

Even more encouraging was the protection observed from the orally administered Low Dose Capsules. The antibody responses induced by oral vaccination depicted in Fig. 7b were produced from serum diluted 1:125 whereas the subcutaneous antibody responses depicted in Fig. 7a were produced from serum diluted 1:2000. Despite being much less concentrated according to the ELISA results, the antibody responses induced by oral vaccination with Low Dose Capsules were considered protective against LeTx challenge at the same serum dilution as subcutaneously vaccinated Low Dose Capsules (Fig. 8, Supplementary Table 2).

Similar to the response from the subcutaneously injected High Dose Capsules, it is also possible that the antibody response due to oral vaccination with Low Dose Capsules had not yet peaked prior to the end of the experiment. In fact, a significant antibody response wasn’t even detected until 31 days post vaccination (Fig. 7b). Given that the gastrointestinal emptying time for a mouse is <24 h^51^, the ELISA data suggest that coated capsules containing Sterne spores may be demonstrating the mucoadhesive properties of alginate by adhering to the intestinal lumen to gradually release their bacterial load^22,52^. This conclusion is also corroborated by the in vitro bacterial release experiment which demonstrated that significant amounts of Sterne spores were still entrapped within High Dose Capsules nearly two months after vaccination (Fig. 5). Continued exposure resulting from extended capsule stability acts as a self-contained booster effect and it is possible that oral vaccination with a higher dose of microencapsulated Sterne spores, or even a booster dose of the same vaccine may further enhance the orally induced immune response.

The findings of this study exemplify the advantages and efficacy of Sterne spore microencapsulation. We have demonstrated that the protein shell is essential for maintaining the controlled release aspects of alginate microcapsules. Our microcapsule formulation is also capable of sustaining Sterne spore viability in an acidic environment and of releasing viable Sterne cells for at least 56 days. Following a single vaccination dose in mice, microencapsulated Sterne spores generated a significant antibody response via subcutaneous, but more impressively, oral vaccination, both of which demonstrated partial to full protection during in vitro LeTx challenge. This immune response can be further enhanced by inoculating a higher bacterial dose with limited adverse effects.

While the results presented here reveal the great potential for this oral vaccine formulation, the majority of wildlife species affected by anthrax are ruminants and thus present further challenges to oral vaccination in the form of three additional stomachs and rumination^53^. Continued research is essential to optimize this vaccine for ruminant species. Future work will involve in vivo studies in a ruminant model to evaluate effective oral vaccination doses and the effect of vaccine boosters. It will also be critical to do an in vivo challenge experiment in a ruminant model to fully demonstrate the protective efficacy of this vaccine. Once the efficacy has been confirmed in a model more closely related to our target wildlife species, we will assess various methods to incorporate this vaccine into a wildlife bait to establish a practical wildlife vaccination method against anthrax. We will also fully explore the potential for environmental contamination with Sterne spores and ensure that no negative consequences will come of a vaccine-laden bait.

In summary, our study reports the achievement of an important first milestone toward the goal of oral vaccination against anthrax. We demonstrate the generation of protective antibody responses from oral vaccination with *B. anthracis* Sterne strain 34F2 spores and with further optimization, we believe this microcapsule formulation has the potential to adapt the Sterne spore for effective oral vaccination of free-ranging livestock and wildlife.

## Methods

### Preparation of Sterne spores

All bacteria used in this experiment were cultured from a vial of the Anthrax Spore Vaccine from Colorado Serum Company (Denver, CO, USA), the North American commercial producer of the Sterne vaccine. The Anthrax Spore Vaccine, which consists of live attenuated *B. anthracis* Sterne strain 34F2 spores in saponin, was used to inoculate a small volume of Luria Broth (LB) and cultured overnight at 37 °C with shaking^12^. The growth was pelleted by centrifugation at 3800 rpm for 15 min, resuspended in LB broth, then plated onto LB agar and incubated at 37 °C for 6 days to sporulate^54–58^. The full bacterial lawns were harvested from the plates and washed repeatedly with sterile water. Remaining vegetative cells were killed by heating at 68 °C for 1 h and removed by filtering through a 3.1 μm filter resulting in a suspension of pure Sterne spores. The final Sterne spore concentration was estimated by plating serial dilutions on LB agar.

### Sterne spore response to simulated gastrointestinal environments

*B. anthracis* Sterne strain 34F2 spores were exposed to simulated gastric or intestinal fluids (GI fluids) to fully comprehend the obstacles to oral vaccination. Simulated gastric fluids consisted of 0.2% (w/v) NaCl and were adjusted to pH 2 and 5 with 1 M HCl to mimic the range of pHs in a non-fasted stomach^33^. Simulated intestinal fluids were 0.68% (w/v) K_2_HPO_4_ adjusted to pH 7 and 8 with 0.2 M NaOH^33^. The pH range convered by the prepared GI fluids was also representative of the environments throughout the ruminant digestive tract where the pH of the rumen is 6.5–7, the reticulum is ~6, the omasum is 4–5 and the abomasum is 2–4^53,59^. A Sterne spore stock solution was prepared at an arbitrary concentration of 3.4 × 10^6^ spores/ml. From this stock solution, 0.2 ml was used to inoculate 6.8 × 10^5^ total Sterne spores into 5 ml of each GI fluid and MOPS Buffer (10 mM MOPS, 0.85% NaCl [pH 7.4]) as a control for future vaccine conditions. The starting spore titer of each inoculated GI fluid was determined by plating serial dilutions on LB agar, then the samples were placed on an orbital shaker at 37 °C. After an overnight incubation, the resulting spore concentration in the GI fluids was determined by plating serial dilutions. Data are reported as the average total recovered colony forming units (CFU) from each buffer.

### Sterne vaccine preparation

The Anthrax Spore Vaccine is distributed by Colorado serum with a recommended 1 ml dose of between 4 × 10^6^ and 6 × 10^6^ viable Sterne spores in saponin for use in cattle, sheep, goats, swine, and horses^12^. This dosage range was simplified to 5 × 10^6^ spores/ml for the purposes of this experiment and was used exactly as received from Colorado Serum Company.

### Microencapsulation of *B. anthracis* Sterne strain 34F2 spores

**Fig. 9:**
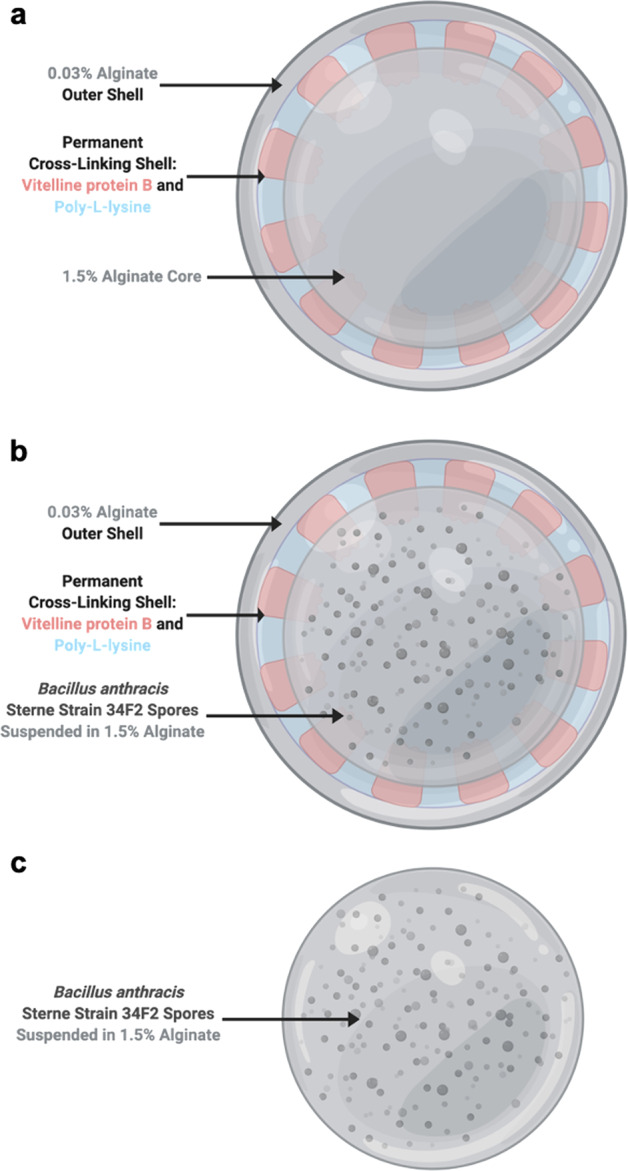
Illustration of microcapsules produced in this study. **a** Capsules without the protein shell loaded with Sterne spores, **b** Empty Capsules coated with the protein shell, **c** Low Dose Capsules loaded with Sterne spores and coated with protein shell. High Dose Capsules (not pictured) were also prepared like the Low Dose Capsules but with a higher amount of Sterne spores. Created with BioRender.com.

Four different microcapsule vaccine formulations with and without the PLL and VpB coating (protein shell) were prepared for the experiments in this study: (i) microcapsules containing 5 × 10^6^ spores/ml without the protein shell, (ii) empty microcapsules with the protein shell (Empty Capsules), (iii) microcapsules containing 5 × 10^6^ spores/ml with the protein shell (Low Dose Capsules), and (iv) microcapsules containing 4 × 10^10^ spores/ml with the protein shell (High Dose Capsules) (Fig. 9).

Microcapsules were prepared similar to previous studies^31^. Sodium alginate (NovaMatrix, Sandvika, Norway) was dissolved in MOPS buffer to a concentration of 1.5% (w/v) alginate. To make the low-dose capsules, 6 × 10^7^ Sterne spores, as determined by the most recent stock titer results, were resuspended in 1 ml of MOPS buffer and then mixed with 5 ml of 1.5% (w/v) alginate solution. Microcapsules were formed using a Nisco Encapsulator VARV1 unit (Nisco Engineering AG, Zurich, Switzerland). The spore + alginate solution was extruded through a 170-μm nozzle, released directly into cross-linking solution (100 mM CaCl_2_, 10 mM MOPS) and stirred for 30 min. The capsules were thoroughly washed with MOPS and then coated with the protein shell by stirring for 30 min in 0.05% PLL and VpB in cross-linking solution. After another washing with MOPS, the capsules received an outer shell of 0.03% (w/v) alginate by mixing for 5 min. Final microcapsule vaccines (Fig. 9) were washed and resuspended 1:1 in MOPS for storage at 4 °C until use. Empty capsules were prepared as above but without any Sterne spores being added to the alginate and high-dose capsules were prepared with a higher amount of Sterne spores added to the alginate. The resulting dose of viable Sterne spores in the microcapsule vaccine was determined by dissolving 1 ml of capsules in 50 mM sodium citrate, 0.45% NaCl, 10 mM MOPS prior to permanent cross-linking with the protein shell^31^. All microcapsule batches were visualized in the brightfield and pixel intensities were measured in ImageJ.

### Characterization of microcapsules in simulated gastrointestinal environments

Microcapsule morphology and bacterial presence within the alginate capsules were visualized with brightfield microscopy. Capsule responses, with and without the protein shell to simulated gastrointestinal fluids (GI fluids) were examined by suspending an aliquot of each capsule formulation in separate vials of the GI fluids. Vials were placed on a tube rocker at 37 °C and samples were collected at 30 and 90 min for imaging on an Olympus CKX41 microscope. Capsule diameters were measured in ImageJ.

### Bacterial release from microcapsules

The bacterial release rates from the microcapsules were examined in vitro by suspending 1 ml of capsules in 9 ml of MOPS buffer and placing the tubes on a rocker at 37 °C^31^. At each sampling time point, the capsules were allowed to settle out of the buffer and then as much of the supernatant as possible was collected without disturbing the capsule pellet. The supernatant was plated on LB agar to estimate the bacterial release since the last time point. Capsules were resuspended in the same volume of MOPS buffer that had been removed and returned to the rocker at 37 °C. Samples were collected every day for 22 days, approximately every other day until day 38 and a final sample was collected at day 56 when the mouse study was terminated. Results are reported in terms of bacterial release per time point versus time.

### Mouse immunizations

Female BALBc/J mice between four and six weeks of age were purchased from The Jackson Laboratory (Bar Harbor, ME, USA). Upon arrival at the animal facility, mice were randomly distributed into six groups of five mice each (Table 1) and allowed to acclimate for at least a week prior to any manipulation. All animal care and experimental procedures complied with all relevant ethical regulations for animal testing and research. The animal experiments conducted in this study received ethical approval from the Texas A&M University Institutional Animal Care and Use Committee (AUP # IACUC 2016-0112).

Mice were inoculated subcutaneously or by oral gavage with 0.2 ml of one of the four prepared vaccines: (i) Empty Capsules, (ii) Sterne vaccine, (iii) Low Dose Capsules, and (iv) High Dose Capsules (Table 1). All mice inoculated with either the Sterne vaccine or Low Dose Capsules received ~1 × 10^6^ spores/mouse while mice inoculated with the High Dose Capsules received ~9 × 10^9^ spores/mouse. The Empty Capsules served as the unvaccinated control. Antibody responses were evaluated in blood samples that were collected 3–7 days prior to vaccination and then every 10–14 days after vaccination for 8 weeks.

### Detection of anthrax-specific antibody levels

Anthrax-specific antibody levels against protective antigen were measured by ELISA^12^. High binding ELISA plates were coated with 100 ng per well of anthrax protective antigen (List Biological Laboratories Inc., Campbell, CA, USA) in carbonate buffer, pH 9.6 and incubated at 37 °C for 1 h, then overnight at 4 °C. The plates were washed 3–5 times with phosphate buffered saline containing 0.5% Tween 20 (PBST). This washing step was repeated between each of the following steps. Next, the plates were blocked for 1 h at 37 °C with 100 μl per well of 1% (w/v) bovine serum albumin in PBST (1% BSA). Serial dilutions of all serum samples were prepared in 1% BSA, loaded 100 μl per well and incubated for 1 h at 37 °C. The secondary antibody, Anti-Mouse IgG (H + L) (SeraCare, Milford, MA, USA, Catalog # 074-1806) was diluted 1:5000 in 1% BSA and loaded 100 μl to a well with a 1 h incubation at 37 °C. TMB/E Substrate (Sigma-Aldrich, St. Louis, MO, USA) was added to each well and the reaction was stopped after 12 min with the addition of 100 μl of 0.5 M H_2_SO_4_. The optical density of all wells was read on a Tecan Infinite F50 Plate Reader at 450 nm. Samples (*n* = 5) from each time point, at each dilution were run in duplicate and experiments were repeated at least three times. Results are reported as average absorbance values for a single dilution for all vaccination groups at each time point. Also reported are the measured antibody titers as the reciprocal of the maximum dilution giving an absorbance greater than two standard deviations above the unvaccinated control.

### Lethal toxin neutralization assays

Toxin neutralization assays were performed to determine the ability of collected serum samples to inhibit the cytotoxicity of anthrax lethal toxin (LeTx) in vitro^14,15,48^. J774A.1 macrophages (ATCC, Manassas, VA, USA, Catalog # TIB-67) were cultured in Dulbecco’s modified eagle medium (DMEM, HyClone) with 10% (w/v) fetal bovine serum (FBS) and 1% (w/v) penicillin. Upon reaching confluency, the cells were harvested and quantified using a hemocytometer, then brought to a final concentration of 5 × 10^4^ cells/ml. Cells were added to a 96-well flat-bottom tissue culture plate at 200 μl/well and incubated overnight at 37 °C in 5% CO_2_. LeTx was prepared by adding lethal factor (List Biological Laboratories Inc., Campbell, CA, USA) and protective antigen (List Biological Laboratories Inc., Campbell, CA, USA) to DMEM containing 10% FBS and no antibiotic at concentrations of 0.25 and 0.1 μg/ml, respectively. The LeTx mixture was used to make serial dilutions of the collected mouse serum samples from each time point on a separate 96-well cell culture plate and then incubated for 1 h at 37 °C, 5% CO_2_. The media was removed from the prepared macrophage plate and replaced with 100 μl/well of the serum LeTx mixture in triplicate. After incubating for 4 h at 37 °C, 5% CO_2_, 10 μl of MTT (3-[4,5-dimethylthiazol-2-yl]-2,5-diphenyltetrazolium bromide; Roche, Basel, Switzerland) was added to each well and incubated for another 4 h at 37 °C, 5% CO_2_. Any remaining metabolically active cells reduced MTT, a yellow tetrazolium salt, to purple formazan crystals using NAD(P)H-dependent oxidoreductase enzymes. The insoluble formazan crystals were dissolved by adding 100 μl of solubilization solution (Roche, Basel, Switzerland) to each well and plates were incubated overnight at 37 °C, 5% CO_2_. The optical density of each well was read at 595 nm using a Tecan Infinite F50 Plate Reader. Cells that were exposed to only LeTx and no serum were used as a positive control. Cells that did not receive any LeTx or serum were used to determine 100% cell viability. The LeTx neutralizing abilities of collected serum samples are reported as average absorbance values for a single dilution for all vaccination groups at each time point from all repetitions of the experiment. Also included are the LeTx neutralizing antibody titers (NT50) reported as the maximum dilution that resulted in over 50% protection which were calculated as ${\mathrm{NT}}50 = \frac{{\left( {\mathrm{mean}\,\mathrm{sample} \,-\, \mathrm{mean}\,\mathrm{LeTx}\,\mathrm{control}} \right)}}{{(\mathrm{mean}\,\mathrm{media}\,\mathrm{control} \,-\, \mathrm{mean}\,\mathrm{LeTx}\,\mathrm{control})}} \times 100.$

### Statistical analysis

Differences between starting and ending titers for Sterne spore responses to GI fluids, and the difference between microcapsule image pixel intensities were determined by two-sided Student’s *t*-tests with *p* < 0.05 considered significant. Across all other experiments, results are expressed as mean values ± standard deviations for all replicates at each time point for each group. Statistical analysis was performed using one-way ANOVA followed by the Tukey–Kramer HSD test with *p* < 0.05 considered significant.

### Reporting summary

Further information on research design is available in the Nature Research Reporting Summary linked to this article.

## Supplementary information

Supplementary Information

Reporting Summary

## Data Availability

All data generated or analyzed during this study are included in this published article (and its supplementary information files).
