# Author Correction: Protective antibody response following oral vaccination with microencapsulated *Bacillus Anthracis* Sterne strain 34F2 spores

**DOI:** 10.1038/s41541-021-00374-9

**Published:** 2021-08-20

**Authors:** Jamie S. Benn, Sankar P. Chaki, Yi Xu, Thomas A. Ficht, Allison C. Rice-Ficht, Walter E. Cook

**Affiliations:** 1 Texas A&M University, Department of Veterinary Pathobiology, College Station, TX 77843 USA; 2 Center for Infectious and Inflammatory Diseases, Institute of Biosciences and Technology, Texas A&M Health Science Center, Houston, TX 77030 USA; 3 Texas A&M Health Science Center, Department of Molecular and Cellular Medicine, College Station, TX 77843 USA

**Keywords:** Bacterial infection, Live attenuated vaccines, Live attenuated vaccines

Correction to: *npj Vaccines* 10.1038/s41541-020-0208-3, published online 10 July 2020

The original version of this Article contained an error in the spelling of the author Jamie S. Benn, which was incorrectly given as Jamie Benn Felix. This has now been corrected in both the PDF and HTML versions of the Article.

