# Supplementary material for: Protective antibody response following oral vaccination with microencapsulated Bacillus Anthracis Sterne strain 34F2 spores
Source: NPJ Vaccines. 2020 Jul 10;5:59. doi: 10.1038/s41541-020-0208-3 (PMC7351773; doi:10.1038/s41541-020-0208-3)
Supplement: Supplementary file 1 — Supplementary Information [file 41541_2020_208_MOESM1_ESM.pdf]

Supplementary Information

Protective Antibody Response Following Oral Vaccination with Microencapsulated *Bacillus anthracis* Sterne strain 34F2 Spores

Jamie Benn Felix*[1], Sankar P. Chaki[1], Yi Xu[2], Thomas A. Ficht[1], Allison C. Rice-Ficht[1,3], Walter E. Cook[1]

[1] Texas A&M University, Department of Veterinary Pathobiology, College Station, TX, 77843 USA
[2] Center for Infectious and Inflammatory Diseases, Institute of Biosciences and Technology, Texas A&M Health Science Center, Houston, TX, 77030 USA
[3] Texas A&M University Health Science Center, Department of Molecular and Cellular Medicine, College Station, TX 77843 USA

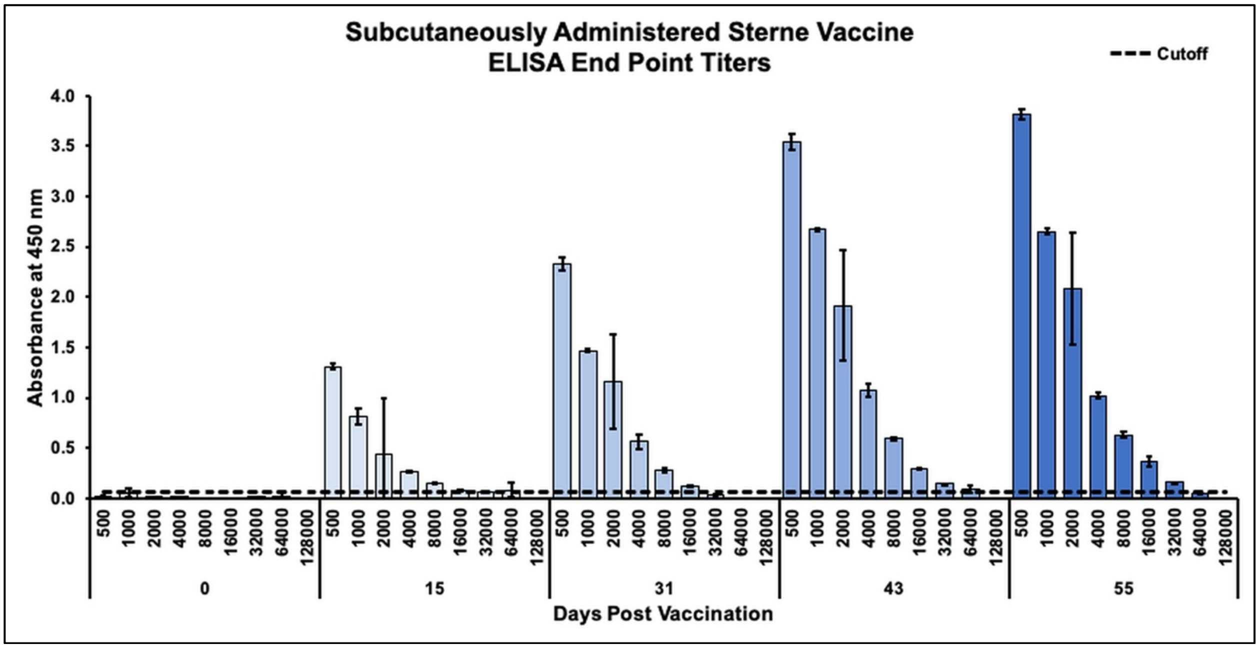

Supplementary Figure 1. End point titer of the antibody response for the subcutaneously administered Sterne Vaccine.
BALBc/J mice were subcutaneously injected with 10^6 unencapsulated *B. anthracis* Sterne strain 34F2 spores. Serum samples were collected at 0, 15, 31, 43- and 55-days post vaccination and analyzed by end point dilution ELISA. Antibody responses are shown as mean absorbances at 450 nm ± standard deviation. The cutoff of antibody detection is approximately two standard deviations above the unvaccinated control.

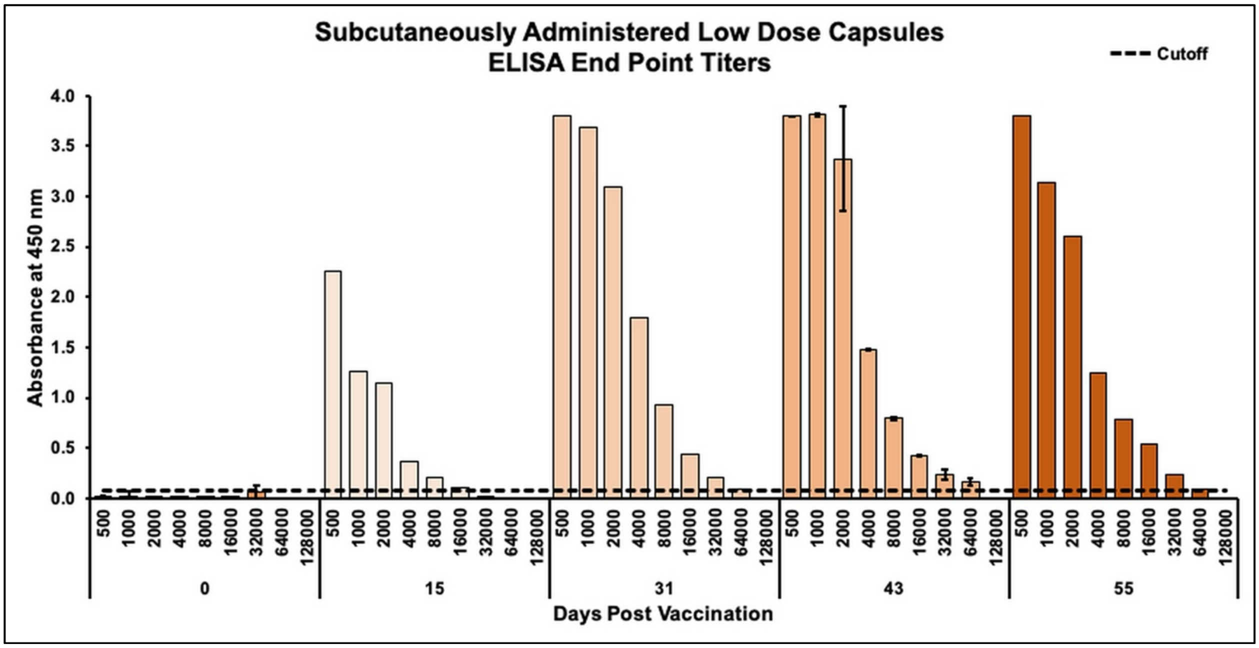

Supplementary Figure 2. End point titer of the antibody response for the subcutaneously administered Low Dose Capsules.

BALBc/J mice were subcutaneously injected with 10^6 encapsulated *B. anthracis* Sterne strain 34F2 spores in Low Dose Capsules. Serum samples were collected at 0, 15, 31, 43- and 55-days post vaccination and analyzed by end point dilution ELISA. Antibody responses are shown as mean absorbances at 450 nm ± standard deviation. The cutoff of antibody detection is approximately two standard deviations above the unvaccinated control.

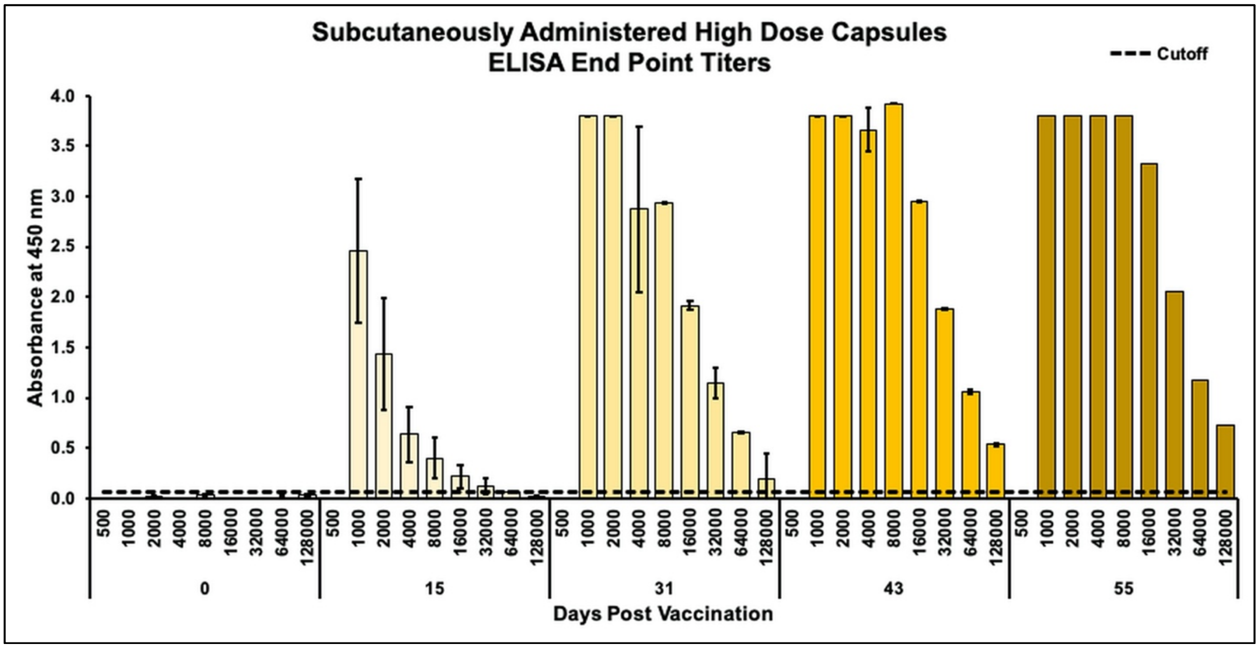

Supplementary Figure 3. End point titer of the antibody response for the subcutaneously administered High Dose Capsules.

BALBc/J mice were subcutaneously injected with 10^9 encapsulated *B. anthracis* Sterne strain 34F2 spores in High Dose Capsules. Serum samples were collected at 0, 15, 31, 43- and 55-days post vaccination and analyzed by end point dilution ELISA. Antibody responses are shown as mean absorbances at 450 nm ± standard deviation. The cutoff of antibody detection is approximately two standard deviations above the unvaccinated control.

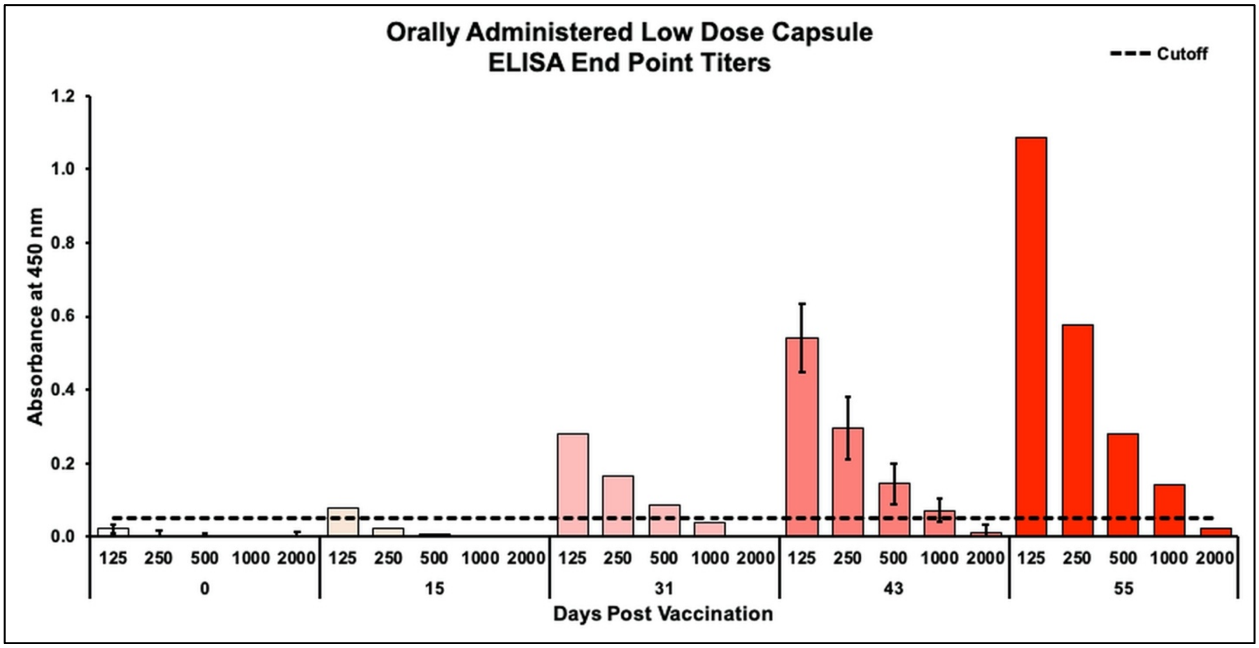

Supplementary Figure 4. End point titer of the antibody response for the orally administered Low Dose Capsules.

BALBc/J mice were orally inoculated with 10^6 encapsulated *B. anthracis* Sterne strain 34F2 spores in Low Dose Capsules. Serum samples were collected at 0, 15, 31, 43- and 55-days post vaccination and analyzed by end point dilution ELISA. Antibody responses are shown as mean absorbances at 450 nm ± standard deviation. The cutoff of antibody detection is approximately two standard deviations above the unvaccinated control.

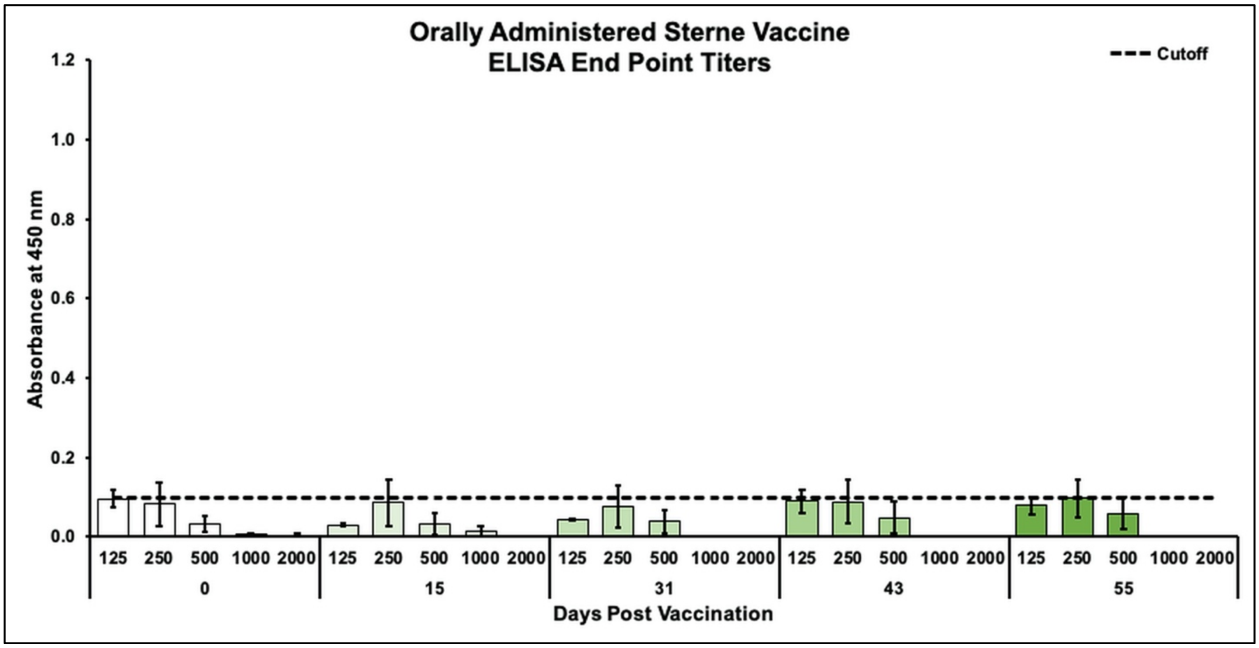

Supplementary Figure 5. End point titer of the antibody response for the orally administered Sterne Vaccine.

BALBc/J mice were orally inoculated with 10^6 unencapsulated *B. anthracis* Sterne strain 34F2 spores. Serum samples were collected at 0, 15, 31, 43- and 55-days post vaccination and analyzed by end point dilution ELISA. Antibody responses are shown as mean absorbances at 450 nm ± standard deviation. The cutoff of antibody detection is approximately two standard deviations above the unvaccinated control.

Supplementary Table 1. Antibody titers against anthrax protective antigen in mouse sera.

Serum antibody titers were determined by end-point dilution ELISA from mice vaccinated subcutaneously and orally with Empty Capsules, the Sterne Vaccine, Low Dose Capsules or High Dose Capsules. BALBc/J mice were either subcutaneously injected or orally inoculated with 10^6 unencapsulated *B. anthracis* Sterne strain 34F2 spores or 10^6 encapsulated Sterne spores in Low Dose Capsules. An additional group of mice were subcutaneously injected with 10^9 encapsulated Sterne spores in High Dose Capsules. Control groups received empty capsules. Serum samples were collected at 0, 15, 31, 43- and 55-days post vaccination and the antibody titer was analyzed by end-point dilution ELISA. The resulting antibody titers are reported as the reciprocal of the maximum dilution giving an absorbance greater than two standard deviations above the unvaccinated control.

**Anthrax Protective Antigen Antibody Titers**						
	**Vaccine**	**Day 0**	**Day 15**	**Day 31**	**Day 43**	**Day 56**
SC	Empty Capsules	ND	ND	ND	ND	ND
SC	Sterne Vaccine	ND	8,000	16,000	32,000	32,000
SC	Low Dose Capsules	ND	8,000	32,000	64,000	32,000
SC	High Dose Capsules	ND	32,000	128,000+	128,000+	128,000+
Oral	Empty Capsules	ND	ND	ND	ND	ND
Oral	Sterne Vaccine	ND	ND	ND	ND	ND
Oral	Low Dose Capsules	ND	125	500	1,000	1,000+

Values reported are reciprocal dilutions. + represents samples that had not yet dropped below the detection limit at the highest dilution made. ND=Not detectable.

Supplementary Table 2. Toxin neutralizing antibody titers against anthrax lethal toxin.

Neutralizing antibody titers against anthrax lethal toxin were determined by toxin neutralization assays with serial serum dilutions from mice vaccinated subcutaneously and orally with Empty Capsules, the Sterne Vaccine, Low Dose Capsules or High Dose Capsules. Serum was collected from mice at 0, 15, 31, 43- and 55-days post subcutaneous or oral vaccination with 10^6 unencapsulated *B. anthracis* Sterne strain 34F2 spores, 10^6 encapsulated Sterne spores in Low Dose Capsules or 10^9 encapsulated Sterne spores in High Dose Capsules. Control groups received Empty Capsules. Diluted serum samples were pre-incubated with LeTx then added to J774A.1 cells and resulting cell viability was assessed with MTT dye. The LeTx neutralizing antibody titers are reported as the reciprocal of the maximum dilution that resulted in over 50% protection which were calculated as: $\boldsymbol{NT50} = \frac{(\boldsymbol{mean\ sample - mean\ LeTx\ control})}{(\boldsymbol{mean\ media\ control - mean\ LeTx\ control})} \times \mathbf{100}$.

Anthrax Lethal Toxin Neutralizing Antibody Titers						
	**Vaccine**	**Day 0**	**Day 15**	**Day 31**	**Day 43**	**Day 56**
SC	Empty Capsules	ND	ND	ND	ND	ND
SC	Sterne Vaccine	ND	200+	50	100+	100+
SC	Low Dose Capsules	ND	200+	200+	200+	200+
SC	High Dose Capsules	ND	800+	800+	800+	800+
Oral	Empty Capsules	ND	ND	ND	ND	ND
Oral	Sterne Vaccine	ND	ND	ND	ND	ND
Oral	Low Dose Capsules	ND	100+	100	50	100+

Values reported are reciprocal dilutions. + represents samples that had not yet dropped below 50% protection at the highest dilution made. ND=Not detectable.
